# Full-Endoscopic Lumbar Discectomy: A Review of the Surgical Techniques, Indications and Anatomical Considerations

**DOI:** 10.3390/jcm14248961

**Published:** 2025-12-18

**Authors:** Stylianos Kapetanakis, Mikail Chatzivasiliadis, Nikolaos Gkantsinikoudis, Konstantinos Pazarlis

**Affiliations:** 1 Spine Department and Deformities, Interbalkan European Medical Center, 57001 Thessaloniki, Greecemikail@chatzi.com (M.C.); nikgkantsinikoudis@gmail.com (N.G.); 2 Academic Orthopaedic Department, Aristotle University of Thessaloniki, 54124 Thessaloniki, Greece; 3 Department of Surgical Sciences, Uppsala University, 75183 Uppsala, Sweden; 4 Aleris Nacka Hospital, 13145 Stockholm, Sweden

**Keywords:** transforaminal endoscopic lumbar discectomy, interlaminar endoscopic lumbar discectomy, unilateral biportal endoscopic lumbar discectomy, lumbar disc herniation, discectomy

## Abstract

Full-endoscopic lumbar discectomy (FELD) has emerged over time as a minimally invasive alternative to conventional microdiscectomy. This narrative review summarizes the available evidence regarding the evolution, indications, techniques, and outcomes of FELD, with a particular focus on how different types of lumbar disc herniations influence the choice of surgical approach. The literature indicates that the transforaminal approach is most suitable for foraminal and upper lumbar disc herniations, whereas the interlaminar approach is preferred for central or migrated L5–S1 herniations due to the larger interlaminar window at this level. Unilateral biportal endoscopy (UBE) provides better flexibility, visualization, and instrument maneuverability, making it particularly useful in certain cases. Reported complication rates remain low overall but vary according to surgical technique and surgeon experience. The learning curve for FELD typically ranges from approximately 20 to over 50 cases, depending on the approach and individual proficiency. Overall, full-endoscopic techniques are redefining the management of lumbar disc herniations by offering less invasive alternatives with favourable clinical outcomes, and their role is expected to expand further as both technology and surgical expertise continue to evolve.

## 1. Introduction

Lumbar disc herniation (LDH) is one of the most common causes of low back pain and lumbar radiculopathy due to nerve root compression [1]. While conservative treatment options are usually sufficient, a portion of patients with persistent symptoms or progressive neurological deficit require surgical intervention [2]. For these patients, open microdiscectomy remains the current gold standard, enabling safe removal of herniated disc under surgical microscope [3]. However, despite its effectiveness, microdiscectomy involves muscle dissection and often even bone removal that may contribute to postoperative pain and, in select cases, segmental instability [3].

Over the past decades, technological advancements have eventually led to the development of full-endoscopic lumbar discectomy (FELD) as an alternative to the traditional microdiscectomy [4]. Depending on the pathology, the procedure can be performed by means of the three primary endoscopic approaches: the transforaminal route, the interlaminar route, or the unilateral biportal route [5].

As these techniques are growingly utilized in the field of spine, there is a lack of integrative reviews comparing their clinical outcomes, complication profiles, anatomical indications, and limitations. Understanding how herniation morphology and anatomical factors influence technique selection is essential. This review aims to collect the current available literature and to evaluate how the different types of LDH influence the selection of FELD techniques, as well as overviewing their current role and potential future applications.

## 2. Anatomy and Pathophysiology of Disc Herniations

The intervertebral discs (IVDs) form the main joints of the spinal column, representing fibrocartilaginous structures that lie between adjacent vertebral bodies [6,7]. IVDs hold almost one-third of the total spinal height, and their primary function is mostly mechanical [6,7,8]. They consist of three distinct components: (1) a central nucleus pulposus (NP), (2) a peripheral annulus fibrosus (AF), and (3) two cartilaginous endplates (CEPs) [6].

The IVDs are mostly avascular, with only small arteries supplying the outer AF and CEPs diffusing the inner AF and NP [7,9]. The outer third of the AF is supplied by the sinuvertebral nerve, which originates from the union of the somatic root of the ventral ramus and autonomic root of the grey ramus communicans [7,10]. On the other hand, NP is not innervated at all [11]. Additionally, the posterior longitudinal ligament (PLL) and outer AF are innervated with nociceptive fibres from the ascending branch of the sinuvertebral nerve, which is associated with chronic low back pain [6].

IVD degeneration is the most common cause of IVD herniation [1]. When the AF is compromised due to this degeneration or trauma, NP protrudes through it leading to a herniation [12]. The majority of the IVD herniations occur posterolaterally, as this area has the least amount of structural support from the longitudinal spinal ligaments and are more susceptible to compressive mechanical injury. This makes the traversing nerve root vulnerable to compression [12]. The exposure of the nerve roots to inflammatory cytokines, such as TNF-α, IL-1β and FGF, contribute to physical pain and radiculopathy [12,13,14]. In addition, FGF and midkine have been shown to recruit macrophages to the site of compression and play a role in both disc resorption and inflammatory response [12,13,14].

From an endoscopic perspective, the anatomical characteristics of the intervertebral foramen and disc space play a decisive role in determining the feasibility and safety of each endoscopic approach. Disc height directly influences the size of Kambin’s triangle, which is the safe working corridor for transforaminal access [15]. This happens due as a reduction in disc height narrows the foraminal vertical diameter and decreases the distance between the exiting nerve root and the superior endplate of the caudal vertebra [15,16]. Consequently, collapsed disc spaces or degenerative foraminal stenosis increase the technical difficulty of transforaminal endoscopy and the risk of exiting nerve irritation during cannula placement [15,16]. In contrast, higher disc spaces provide a larger transforaminal window and facilitate more favourable needle angulation.

At the lumbosacral junction, anatomic constraints influence approach selection. A high iliac crest increased sagittal facet joint orientation, and hypertrophic superior articular processes (SAP) significantly restrict the lateral trajectory required for transforaminal access at L5–S1, thereby reducing the working space available for endoscopic instruments [17]. These anatomical limitations favour an interlaminar or biportal posterior approach, where the naturally wide L5–S1 interlaminar window provides a direct and spacious corridor to the herniated fragment without the need to traverse the foramen [17].

These anatomical constraints not only determine the natural pathways available for endoscopic access but also shape the radiological appearance and spatial distribution patterns of lumbar disc herniations. It is essential to understanding the structural relationships when interpreting LDH morphology and selecting the most appropriate endoscopic corridor, as discussed in the following section.

## 3. Radiological Stages of Disc Herniations

Given the clinical impact of LDH, a standardized approach to classifying disc pathology has been developed, improving decision making [18]. The morphological disc herniation types along with their definition and clinical considerations according to the North American Spine Society (NASS) nomenclature are summarized in Table 1 [19]. However, clinicians should always consider that surgical intervention is also associated with the topography of LDH, rather than radiologic morphology only. Beyond descriptive radiologic categorization, herniation morphology has practical implications for endoscopic management. Extrusions and sequestrations, particularly those with cranial or caudal migration, may require wider surgical corridors and demonstrate variable success across approaches [20,21]. On the other hand, protrusions often require less extensive decompression when surgery is indicated [20,21].

**Figure 1:**
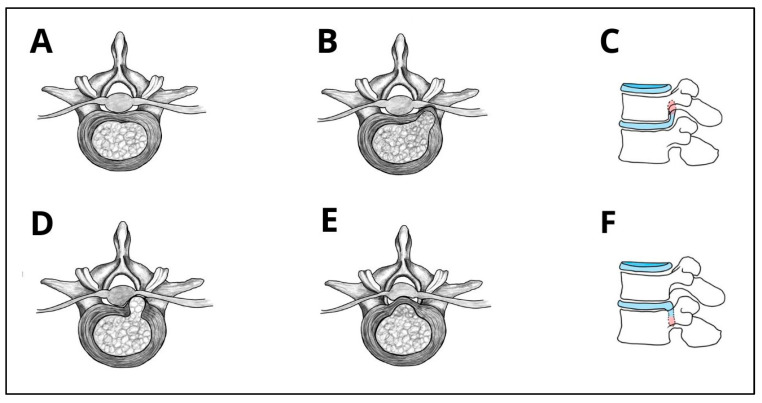
Schematic overview of LDH types and migration patterns. (**A**) Normal vertebra without herniation; (**B**) foraminal LDH; (**C**) cranially migrated disc herniation; (**D**) paracentral herniation; (**E**) central herniation; (**F**) caudally migrated disc herniation.

The anatomical location of the disc material also plays a significant role in surgical planning. Therefore, LDHs can be classified based on their position relative to the spinal canal and neural elements. Table 2 describes the preferred endoscopic approaches for each herniation type based on anatomical location, an element of crucial importance in preoperative planning [21,22,23,24,25,26,27,28,29,30,31,32,33,34,35,36,37,38]. These anatomical distinctions directly influence the available working corridor, determine the likelihood of complete decompression, and affect the risk of reoperation, particularly in foraminal and cranially migrated herniations where access may be limited. Additionally, Figure 1 provides schematic representations of the most common LDH types.

## 4. Transforaminal Endoscopic Lumbar Discectomy

### 4.1. General Overview

Before moving on to the specifics of each technique, it is important to note that such minimally invasive procedures are generally indicated in patients with persistent radicular symptoms who fail to respond to adequate conservative therapy. TELD is indicated in patients that present with unilateral radiculopathy, positive nerve root tension sign, foraminal, paracentral, or migrating disc herniations which, especially, are considered for this type of surgery (see Table 2) [26]. In addition, TELD approach is most appropriate in the upper lumbar levels due to larger foraminal dimensions [26].

**Figure 2:**
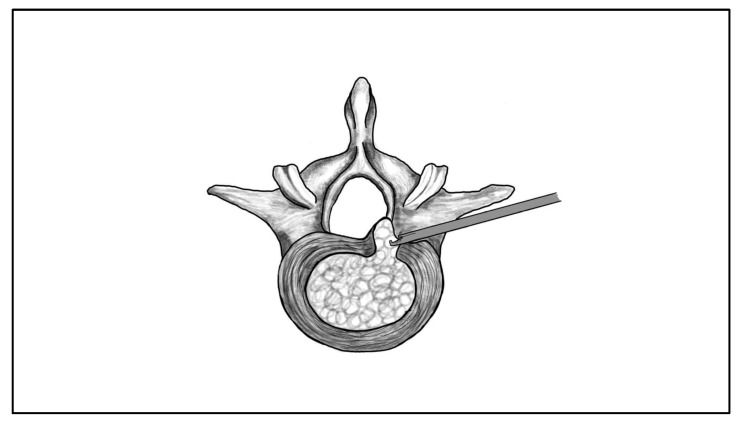
Illustration of the transforaminal endoscopic lumbar discectomy (TELD) approach. The endoscope and working instruments are inserted through Kambin’s triangle.

**Figure 3:**
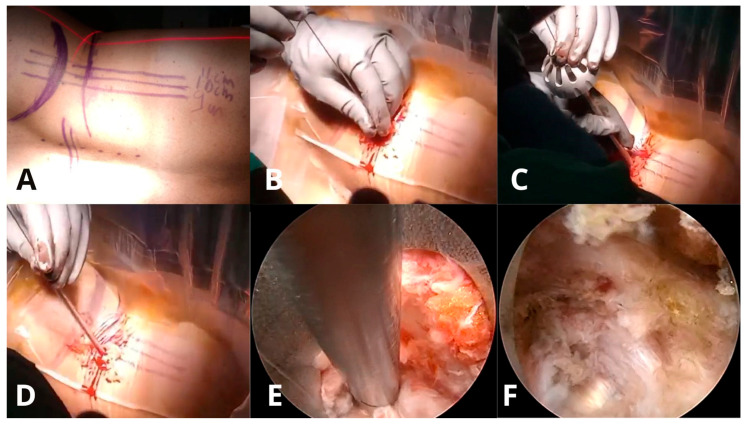
Step-by-step intraoperative sequence of TELD. (**A**) Patient positioning and skin marking in lateral decubitus position with planned entry trajectory. (**B**) Insertion of the K-wire at the predetermined entry point under fluoroscopic guidance. (**C**) Sequential foraminal dilation using reamers to expand the working corridor. (**D**) Placement of the bevelled working cannula through the soft tissue corridor toward the disc space. (**E**) Endoscopic view showing excision of herniated disc material using grasping forceps. (**F**) Final endoscopic view demonstrating a pulsatile traversing nerve root after successful decompression, visualized as a white band, indicating the primary endpoint of adequate neural release.

However, the feasibility of TELD largely depends on the size of Kambin’s triangle, which depends on disc height, foraminal width, and the relationship between the exiting nerve root and the superior articular process [15]. Choi et al. demonstrated that a reduced “nerve root–facet distance” is associated with higher risk of exiting nerve irritation during transforaminal access, which shows the importance of the preoperative assessment of foraminal dimensions [27]. Their data implied that when this distance is approximately 5 mm or less, the risk of exiting nerve irritation increases substantially [27]. In such cases, alternative approaches or planned foraminoplasty should be considered to avoid iatrogenic root compression [27]. Anatomical studies further show that disc space collapse or degenerative foraminal stenosis narrows the available corridor, whereas high discs provide greater maneuverability for instrument passage [28,29]. Figure 2 provides an illustration of the TELD approach. Working corridor of TELD is schematically depicted in Figure 3.

At the L5–S1 level, TELD may be limited by a high iliac crest, transverse process height, or steep facet orientation, which can obstruct the ideal trajectory and reduce the safety margin for cannula placement [30]. Sousa et al. mentioned that the use of foraminoplasty or adjusted trajectories did have successful outcomes [31]. Herniation morphology also affects the expected outcomes. Intraforaminal and high-grade migrated herniations demonstrate lower rates of complete decompression and a higher likelihood of revision compared with standard paracentral pathology [32]. In addition, clinical outcome studies demonstrate that foraminal, far-lateral, and highly migrated herniations are associated with higher rates of incomplete decompression and revision [32]. In a 5-year series of 204 TELD patients, foraminal and far-lateral herniations showed significantly lower excellent/good outcomes and higher postoperative symptom persistence compared with paracentral herniations, mostly due to the restricted working space adjacent to the dorsal root ganglion [32].

TELD shares the general contraindications applicable to all lumbar endoscopic procedures, such as spondylolisthesis, spinal tumours, infection, and vertebral fractures, while technique-specific limitations include free sequestered fragments [26].

### 4.2. Surgical Technique and Anatomical Landmarks

TELD is a minimally invasive procedure that can be performed under local anesthesia so that the patient can be neurologically monitored intraoperatively [39]. There are two primary phases in this technique: (1) percutaneous access through the intervertebral foramen under fluoroscopic guidance, and (2) endoscopic decompression of the IVD herniation with real-time visualization [39]. Preoperative planning is essential, as safe access depends on understanding patient-specific foraminal anatomy and the dimensions of Kambin’s triangle.

MRI, X-rays, and Computed Tomography (CT) scans are used to localize the herniation, determine the optimal skin entry point, and evaluate factors such as disc height, foraminal width, and iliac crest level at L5–S1 that may influence the working corridor [25,40,41,42]. Preoperative imaging is also crucial for trajectory planning as cranially migrated, foraminal, or far-lateral herniations often require adjustments in needle angulation to safely reach the fragment.

The patient lies prone on a radiolucent table. Alternatively, the lateral decubitus position may be adopted, with hip and knee in flexion to enlarge dimensions of the foraminal space [25,26,41]. Based on the anatomy, the entry point is usually 8–14 cm lateral to the midline [25,37,42]. The optimal trajectory varies with herniation morphology and localization. Paracentral herniations are approached with a shallow cranio-caudal angle toward the posterior third of the disc. Cranially or caudally migrated herniations are approached with a corresponding cranial or caudal adjustment in the needle trajectory.

Intra- and extraforaminal herniations require a more medial entry on anteroposterior (AP) imaging to align directly with the foramen.

The working corridor provides access to Kambin’s triangle, the safe transforaminal window bordered by the exiting nerve root, the superior endplate of the caudal vertebra, and the SAP [4,37]. On AP fluoroscopy, the needle should track between the pedicle borders to avoid the exiting root, while the lateral view confirms depth and cranio-caudal alignment toward the posterior disc margin, with minor angle adjustments made according to the location and migration pattern of the herniated fragment.

Under fluoroscopic guidance, an 18-gauge spinal needle is inserted along the pre-planned trajectory. On AP imaging, the needle tip should lie between the medial and lateral pedicle borders, which ensures that the pathway stays lateral to the traversing root while avoiding the exiting root situated just inferior to the pedicle [25,42]. A guidewire is then inserted through the needle which is then followed by a stab incision so that a cannulated dilator can be introduced to gently dilate the soft tissues [41,42]. This is what ultimately establishes the working field [41,42]. In the outside-in variant of TELD, progressively larger reamers are being introduced over the guidewire to decompress the foramen before accessing the IVD, with diameters ranging from 4 mm to 9 mm [39]. This step is particularly important in patients with foraminal stenosis or collapsed disc height where Kambin’s triangle is reduced [39]. Preoperative studying of the foraminal dimensions with the use of MRI and CT is extremely important when selecting the range of reamers in every patient.

Once foraminal decompression is achieved during this version of the technique, the obturator is introduced [26]. A bevelled working cannula is placed over the obturator and positioned on the lateral aspect of the AF, which is fluoroscopically confirmed [42]. Finally, the endoscope is introduced along with continuous irrigation of the working channel. At this phase, it is crucial to first identify the anatomical landmarks: the SAP, the caudal pedicle, the exiting and traverse nerve roots, the AF and the herniated disc material [25,41]. Discectomy can be safely performed at this stage. The procedure is finished once the surgeon visualizes the dural sac and there is free mobilization of the nerve root [25,26,42].

### 4.3. Complications, Technical Risks, and Learning Curve

Following the technical description of TELD, it is important to be aware of the potential complications and the associated learning curve that comes with it. A study by Fan et al. reported the total complication rate to be around 9.76% [43]. The most commonly reported complications included dysesthesia (1.46%), radicular pain (1.20%), dural tears (1.9%), recurrent LDH (2.3%), and persistent postoperative pain (3.79%) [43,44,45]. Postoperative dysesthesia and radicular irritation are most commonly related to manipulation within a narrow Kambin’s triangle, where the exiting nerve root and dorsal root ganglion lie in close proximity to the working cannula [44]. Dural tears occur more often in cases with migrated fragments because the traversing root and the anteromedial dura are displaced by herniated material, reducing the available working margin [39,44]. Patients with highly migrated herniations or foraminal stenosis also featured a higher incidence of incomplete decompression where reoperation was needed in 2–15% of cases [44]. Less common complications were infection (0.3%), epidural hematoma formation (0.04%), postoperative neurologic decline, pedicle fractures, urinary retention and intracranial hypertension [43,45]. In the systematic review by Ju et al. no major perioperative complications were found. The latter supports that TELD can be safely performed with the appropriate surgical expertise [45].

Competency in TELD was reported after 7 to 72 cases, with a median learning curve of around 20 cases [46]. Operative time was decreased from 114 min to 80 min after the first 20 cases [46]. Another study identified a threshold of 35 cases, which also reported a 26.3% reduction in operative duration [46,47]. These findings suggest that both efficiency and complication rates improve markedly with increasing surgeon familiarity, particularly with regard to anatomical orientation and fluoroscopic trajectory planning.

### 4.4. Perioperative and Health-Economic Considerations

Economic evaluations comparing transforaminal endoscopic discectomy with open microdiscectomy consistently show favourable societal cost-effectiveness for endoscopy. In a large RCT-based analysis, Gadjradj et al. demonstrated that transforaminal endoscopy offers lower overall societal costs and improved quality-adjusted life-years (QALYs) despite slightly higher procedural expenses [48].

In terms of perioperative factors, radiation exposure is higher in transforaminal approaches; Zhou et al. reported significantly greater fluoroscopy use in TELD than IELD, reflecting the need for repeated AP and lateral confirmation within Kambin’s triangle [49]. However, this limitation can be modified by changing certain factors. Yao et al. showed that navigation-assisted TELD reduces fluoroscopic shots and cumulative radiation dose, which shows that adjunctive technologies can minimize radiation burden [50].

Functional recovery is another area where endoscopic techniques demonstrate consistent advantages. Lewandrowski et al. reported rapid return-to-work (RTW) trajectories after outpatient endoscopic transforaminal decompression, with over 90% of patients resuming work early in the postoperative period [51]. Similarly, Derman et al. found a median RTW of 16 days in a large United States (U.S.) cohort of lumbar endoscopic spinal surgery patients, with more than 80% returning to work within 90 days [52].

## 5. Interlaminar Endoscopic Lumbar Discectomy

### 5.1. General Overview

The IELD technique has initially been developed to treat central and paracentral herniations at L5–S1 level due to the wide interlaminar window [38,53,54]. The wide interlaminar space provides a direct posterior epidural route, which facilitates access to cranially and caudally migrated disc fragments that often lie beyond the reach of a transforaminal working corridor [53,54]. Nevertheless, over time, this technique started being used more broadly in cases of large, migrated disc herniations, and even in foraminal and extraforaminal lesions via modified approaches [38,53,54]. Whenever TELD is restricted due to low intertransverse space, overlapping facet joints, or high iliac crests, the IELD technique may be effectively recruited [38,53,54]. In such anatomical scenarios, IELD bypasses foraminal constraints by accessing the epidural space posteriorly, making it less affected by disc height, facet orientation, or iliac crest height compared with TELD.

**Figure 4:**
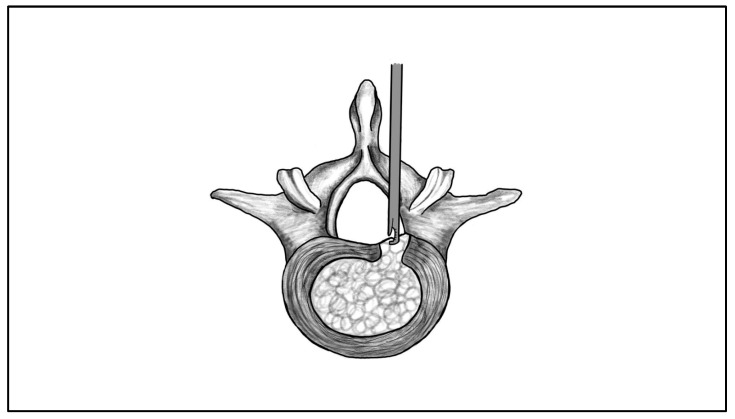
Illustration of the interlaminar endoscopic lumbar discectomy (IELD) approach. The endoscope and working instruments are inserted through the interlaminar space.

Clinical series of IELD, particularly for central, paracentral, or far-migrated lumbar disc herniations, report favourable outcomes with good or excellent results in approximately 90% of cases at 1–2 years follow-up [38]. In patients with far-migrated fragments, IELD achieves satisfactory leg pain relief and functional improvement comparable to conventional microscopic interlaminar discectomy [38]. However, most IELD outcome studies report aggregated results without separating cases by herniation subtype (e.g., central, paracentral, foraminal, extraforaminal, or migrated). As a result, the literature provides limited morphology-specific outcome data for IELD, and direct comparisons between subtypes remain difficult to interpret. Working corridor of IELD is schematically depicted in Figure 4.

IELD is indicated in patients who are unresponsive to conservative treatment and have large disc material that occupy more than 50% of the spinal canal with high grade caudal or cranial migration [22,53]. IELD is contraindicated when there is severe scoliosis or calcified disc herniations with adhesions to AF [22,53]. In addition, at upper lumbar levels the interlaminar window is narrower which also affects IELD application.

### 5.2. Surgical Technique and Anatomical Landmarks

IELD is a minimally invasive technique that allows posterior access to the spinal canal through the interlaminar window [22,25,38,53]. In contrast to TELD, IELD is usually performed under general anesthesia [34,53]. Intraoperative fluoroscopy is used to localize the affected levels and to guide the entire surgical procedure [22,25,38,53]. Anatomical landmarks, such as the ligamentum flavum, laminae, and superior articular process, are essential for safe orientation during cannula placement [22,25,38]. Unlike the transforaminal approaches, IELD requires working in close proximity to the dural sac and traversing nerve root, which makes it more important to precisely identify the anatomy and handle tissue carefully [38,53].

The skin entry point is planned over the interlaminar window, with its medial–lateral position adjusted according to the width of the space and the morphology of the adjacent laminae [25,38,53]. At L5–S1, the large interlaminar window allows a relatively direct trajectory, whereas at upper lumbar levels a more oblique approach is required to reach the inferomedial margin of the rostral lamina due to the narrower interlaminar height and disc position [38,53]. The working trajectory is also subtly adjusted according to herniation morphology. Cranially migrated fragments require the cannula to be angled slightly superiorly, whereas caudally migrated fragments require a more caudal tilt to expose the distal epidural space. These differences are anatomical variations, including laminar overlap, facet joint inclination, and the thickness of the ligamentum flavum, which together determine the available working corridor. Following a small incision, the deep fascia is opened, sequential dilators are then advanced toward the target area under fluoroscopic guidance, and a bevelled working cannula is inserted once the correct trajectory is confirmed [22,25]. In cases of severe degeneration, a partial hemilaminotomy may be required to widen the working area [22,25]. Upper lumbar levels often present additional difficulty due to the narrower interlaminar window and that the ligamentum flavum may be partially obscured by overlapping laminae [54]. At L5–S1 level, there is sufficient space for direct access without the need to resect bone [22,54]. Flavotomy and partial flavectomy is then performed to enter the epidural space. This may be facilitated with the use of saline pressure in the working cannula [54]. Although several techniques exist, a longitudinal incision parallel to the fibres of the ligamentum flavum is most commonly used because it provides controlled expansion while minimizing the risk of dural injury [54]. The two-layered structure of the ligamentum flavum and its variable thickness across levels make this step particularly critical, as inadvertent penetration of the inner layer may directly expose or injure the dural sac.

Once the interlaminar window is exposed, the outer layer of the ligamentum flavum is separated from the underlying lamina and facet surface, allowing visualization of the thicker inner layer that directly overlies the dural sac [25,53]. Because the inner layer forms the final barrier before the epidural space, its opening must be performed in a controlled fashion to avoid dural injury, particularly at levels where the flavum is thin or partially deficient [22,53]. Endoscopic orientation is maintained by identifying consistent landmarks such as the lamina, superior and inferior articular processes, the facet capsule, and the remaining ligamentum flavum fibres [22,25,53].

**Figure 5:**
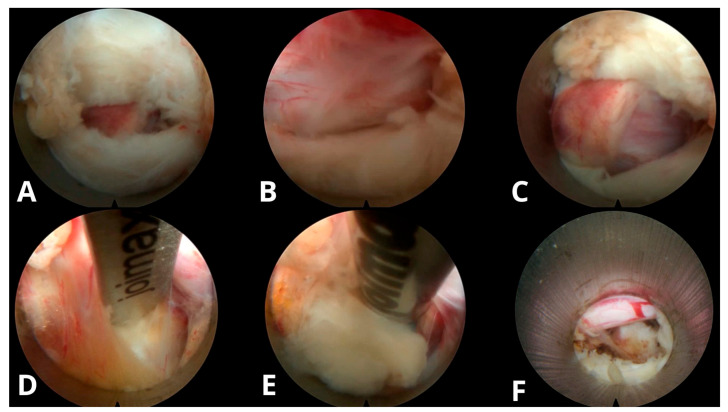
Step-by-step intraoperative endoscopic views during an IELD at L5–S1 at our outpatient clinic. (**A**) The procedure begins with the initial flavotomy, where the outer layer of the ligamentum flavum is opened to access the deeper layers. (**B**) Further dissection allows entry into the epidural space, exposing the neural structures. (**C**) The working channel is advanced toward the epidural space. (**D**) The traversing nerve root is mobilized, revealing the displaced disc material beneath it. (**E**) A radiofrequency probe is used to safely release the LDH from the annulus. (**F**) Final inspection after disc removal confirms full decompression of the nerve root and restoration of the epidural space.

During decompression, the orientation of the working cannula is a key protective maneuver. The bevelled opening is initially rotated toward the neural elements, so that the bevel functions as a shield that gently displaces and protects the dura and traversing nerve root. After safe entry into the epidural space is confirmed, the cannula is intermittently derotated to reduce focal pressure on neural structures and restore a neutral working angle. This sequence is especially important in stenotic canals or when large, migrated fragments restrict the available epidural workspace, as small adjustments in cannula position can significantly influence nerve root tolerance [53]. In cases of contralateral recess stenosis or bilateral symptoms and pathology, the endoscope may be angled across the midline to achieve “over-the-top” decompression while preserving contralateral bony elements [22,25]. An illustration of the intraoperative steps during IELD is shown in Figure 5. The anatomical constraints and technical steps outlined above directly influence the complication profile of IELD.

### 5.3. Complications, Technical Risks, and Learning Curve

IELD is associated with certain risks and potential complications. The overall complication rate has been reported to be around 3.4%, which has been depicted to be lower than TELD in specific reports [44,55]. The most common complications included dural tears (2.19%), recurrent disc herniation (3.5%), and dysesthesia (1.3–3.1%) [39,50]. Less commonly reported complications are epidural hematoma (0.06–0.4%), infection (0.1%), and incomplete decompression (<5%) [44,55].

Compared to the transforaminal approach, IELD carries a lower risk of exiting nerve irritation but a slightly higher risk of dural injury due to the intradural-adjacent working corridor. The competency in the IELD technique is achieved after around 50 to 60 cases, making it harder to master than the TELD approach [56,57]. Other studies suggest that proficiency is reached after around 51 cases [56,57]. The steep learning curve is largely attributed to the need for precise manipulation adjacent to the dural sac, the reliance on two-dimensional endoscopic depth perception, and the limited working space at upper lumbar levels. The operative time has also been shown to decrease significantly, from an average of 90.2 min to 71.5 min [56,57].

### 5.4. Perioperative and Health-Economic Considerations

Economic evaluations comparing IELD with conventional microsurgical approaches show better cost-utility profiles for endoscopy. In a comparative economic analysis, Choi et al. reported that endoscopic discectomy, like IELD, reduce total hospital-related costs by 23.1% and QALYs with values of 0.211 for IELD versus 0.186 for microdiscectomy, largely due to shorter hospitalization and faster postoperative recovery [58].

In terms of perioperative factors, radiation exposure is lower in interlaminar approaches. Amato et al. demonstrated that the interlaminar approach requires less radiation than the transforaminal approach, reporting a mean exposure of 8.37 ± 4.21 mGy for IELD versus 28.92 ± 7.56 mGy for TELD, along with shorter fluoroscopy times (11.2 ± 5.5 s vs. 42 ± 16.6 s) [59]. This is partially due to the reduced need for repeated AP–lateral confirmation during access planning [59].

Functional recovery is likewise favourable in IELD. In a multicenter analysis of 545 IELD patients, Wasinpongwanich et al. reported that most patients experienced early mobilization within 24 h, with rapid improvement in leg pain and disability supporting early return to activity [60]. Similarly, Derman et al. found a median RTW of 16 days with more than 80% of patients returning to work within 90 days [52].

## 6. Unilateral Biportal Endoscopic Lumbar Discectomy

### 6.1. General Overview

**Figure 6:**
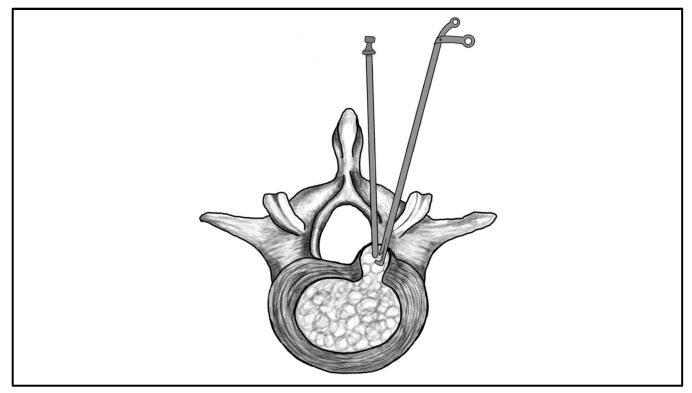
Illustration of the unilateral biportal endoscopic lumbar discectomy (UBE-LD) technique. Two independent portals are used: one for endoscopic visualization and one for instrumentation.

Unilateral biportal endoscopic lumbar discectomy (UBE-LD) is a minimally invasive surgical technique that makes use of two portals, one for visualization and the other for instrument manipulation [61,62,63]. Compared to the uniportal techniques that were previously mentioned, this allows for a wider range of view, as well as easier adaptation of the surgical instruments [23,61,63]. This approach has been clinically used in various types of discs herniations, including central, paracentral, foraminal, extraforaminal and even migrated pathologies [23,34,61,62,63]. In addition, it can provide bilateral decompression via a unilateral approach, while still preserving the facet joint and lamina [61,62,63]. Figure 6 illustrates the UBE-LD method.

Furthermore, this approach is particularly preferred in complex high-migrated cases, or multilevel stenosis and revision cases where the other endoscopic techniques are particularly limited [62,63]. UBE achieves higher rates of complete decompression in high-grade migrated herniations than uniportal approaches, largely due to its broader field of view and greater instrument-handling freedom [62,63]. However, this technique is also contraindicated in specific cases, like segmental instability or severe spondylolisthesis [62,63]. Compared to TELD and IELD, some authors suggest that UBE may allow greater dural-sac expansion and comparable decompression in complex or multilevel stenosis, which could be advantageous in selected migrated or stenotic cases [64,65]. However, other comparative studies show similar functional outcomes and even shorter hospital stays with uniportal techniques, indicating no definitive superiority of UBE for all patients [64,65]. Additionally, even though UBE has been gaining a lot of popularity in the past years, surgeons must stay on the lookout for complications like thermal nerve injury, increased intracranial pressure, dural tears, and instability due to excessive bone resection [63].

### 6.2. Surgical Technique and Anatomical Landmarks

Even though it is possible for the UBE-LD technique to be performed under local or spinal anesthesia, it is generally performed under general anesthesia to make sure that the muscles are fully relaxed and patient is stable [23,34,61,62,63]. As with other posterior endoscopic approaches performed in the prone position, the patient is placed onto a radiolucent table using the Wilson frame to reduce lordosis and widen the working space [61]. Regardless of the approach used, it is important to minimize abdominal pressure during the surgery as this could lead to epidural venous bleeding, so the patients should be elevated with padded supports [61,62]. Additionally, the mean arterial pressure should be kept below 80 mmHg to reduce bleeding risk [61]. Portal placement is also guided by fluoroscopy with AP and lateral views [61]. As the visualization and working portals originate from separate trajectories, UBE relies heavily on triangulation. Thus, the cranio-caudal spacing and medial–lateral alignment of the portals determine the operative angles available for laminotomy, flavectomy, and fragment removal. Small variations in portal position can significantly alter access to the lateral recess or migrated fragments, making preoperative planning essential.

There are three main UBE-LD surgical approaches that are used depending on the location of the herniation: (1) the interlaminar approach for central and paracentral herniations, (2) the contralateral approach for opposite lateral recess stenosis or hidden zone herniations, and (3) the paraspinal approach for foraminal and extraforaminal disc herniations by targeting the lateral facet and transverse process [61,62]. The modularity is one of the key advantages of the biportal system, as each approach leverages different anatomical corridors (interlaminar, epidural midline, or paraspinal gutters) to reach pathology that may be difficult to access with uniportal endoscopy.

In the standard interlaminar approach, there are two incisions made 1 cm caudally and cranially the medial pedicle line of the affected disc level with an additional 2–3 cm spacing between them for better triangulation [34,61,62]. The portals are aligned with the lower margin of the cranial pedicle and at the midpoint of the caudal pedicle depending on the location of the disc herniation [61]. For example, foraminal or upward migrated herniations require a more caudal placement, and downward migration requires a more upward placement [61]. On the other hand, in paraspinal approaches the skin incisions are made on the tips of the spinous processes, 1–1.5 cm lateral to the pedicle to target the foramen in the lateral view and the docking point is aligned with the lateral border of the SAP or isthmus, depending on the level [61,62]. Additionally, placement of the portals is often difficult at the L5–S1 level due to the iliac crest being in the way and the facet joints being coronally oriented [61]. For example, when operating on the left side of this level, the working portal may be required to be placed 1 cm medial to the routine portal to bypass the iliac crest [61]. The level-dependent adjustments highlight the importance of understanding facet morphology, laminar width, and iliac crest height, as inadequate portal adjustment can restrict triangulation and limit exposure. Once both portals are placed, the instruments and endoscope are triangulated to check if they are correctly angled within the interlaminar or paraspinal space [61,62].

Once correct placement is confirmed, a working space has to be created between the portals with the use of serial dilators [23,34]. These are inserted gently to position the multifidus muscle away from the lamina and facet joint, so the interlaminar or paraspinal space is opened depending on the approach used [62]. The viewing portal is subsequently established, and an endoscope (zero or thirty degrees, depending on localization of pathology being treated) is inserted at set to the isthmus [61,62]. Ideally, this is performed with a low-pressure pump under 30 mmHg or via gravity to avoid neurological complications [61,62]. A clear visual field is established by removing soft tissue and fat by using a radiofrequency probe and shaver, identifying landmarks such as the spinolaminar junction and the ligamentum flavum [61,62]. As the working portal is independent, UBE allows instruments to approach the LF and lamina from a more favourable angle compared with uniportal systems, especially in stenotic lateral recesses.

Laminotomy starts at the lower edge of the cranial lamina by using a drill that thins the bone until the LF is visible [61]. The lateral side of the dural sac and traversing nerve root are visualized and discectomy after proper nerve root medialization may be effectively performed. In the contralateral approach, the approach to laminotomy and flavectomy is more medial than the rest, requiring a more extensive resection of bone to reach the dural sac.

### 6.3. Complications, Technical Risks, and Learning Curve

The total complication rate in UBE-LD ranges from 5% to 6.7% across multiple studies [39,59]. The most reported complications are dural tears (2–4.1%), epidural hematoma (1.9%), and recurrent disc herniation (<5%) [44,66]. Less commonly reported complications include transient paresthesia (0.14%) and infection (<0.5%) [42,62]. Higher rates of dural tears compared to TELD and IELD may be related to the absence of a rigid working cannula, which allows greater soft-tissue mobility but also exposes the dura more during laminotomy. On the other hand, UBE demonstrates a lower incidence of postoperative dysesthesia because the exiting nerve root and dorsal root ganglion are not subjected to prolonged cannula pressure.

When comparing UBE-LD to the other uniportal endoscopic techniques, a slightly higher rate of dural injuries and postoperative hematomas may be encountered. However, this procedure is related to a lower incidence of postoperative dysesthesia. Considering learning curve, competency in UBE-LD is achieved after 32 to 54 cases, which obviously depends on the surgeon’s prior experience [67,68]. Peng et al. reported proficiency after around 32.2 cases, while Xu et al. put the threshold at 54 cases [67,68]. Table 3 provides a comparing overview and the differences between TELD, IELD, and UBE-LD [56,57,61,69,70,71,72,73,74,75,76,77,78].

### 6.4. Perioperative and Health-Economic Considerations

Economic evaluations specifically comparing UBE-LD with other endoscopic approaches are limited, but emerging evidence suggests meaningful cost differences. In a recent DRG-based retrospective cohort study of 364 patients, Chen et al. found that UBE was significantly more expensive than TELD. Cost drivers included higher anesthesia, treatment, medication, and consumables fees in the UBE group, despite comparable clinical outcomes including recurrence (12.9% vs. 13.8%) and reoperation rates (0.8% vs. 1.7%) after matching [79].

Regarding radiation, Merter et al. prospectively compared UBE, PLED and tubular discectomy, reporting mean fluoroscopy times of 19.3 s for UBE, 34.9 s for PELD, and 4.6 s for tubular discectomy. Thus, UBE carries an intermediate radiation burden, lower than posterolateral PELD but higher than tubular discectomy [80].

## 7. Conclusions

This review highlights how the three major full-endoscopic lumbar discectomy techniques offer distinct anatomical corridors and technical advantages that make them suitable for different patterns of disc pathology. TELD provides an effective posterolateral route for paracentral, foraminal, and upper lumbar herniations when Kambin’s triangle gives sufficient working space. In contrast, IELD offers direct posterior access and is particularly useful for large or cranially/caudally migrated fragments at L5–S1. UBE further increases surgical flexibility and is especially advantageous in stenotic, multilevel, or revision cases that require broader decompression through its two-portal triangulation. These distinctions show that clinical outcomes depend not only on surgeon’s experience but also on careful preoperative assessment of foraminal dimensions, disc morphology, and facet or iliac crest constraints. Future advances in optics, navigation, and instrument design may further refine these techniques and broaden their applicability. Thoughtful selection among these complementary approaches will remain essential as endoscopic spine surgery continues to evolve within modern minimally invasive practice.

## Figures and Tables

**Table 1:** Summary of morphological types of LDH. Including their definitions and relevant clinical considerations, adapted from the NASS 2014 lumbar disc nomenclature version 2.0 [19].

Morphological Type	Definition	Clinical Considerations
Protrusion	Localized displacement where the width of herniation is smaller than the width of the base at the disc margin	Often asymptomatic or mildly symptomatic; usually responds well to conservative treatment; persistent bulging may lead to poorer conservative outcomes in some cases.
Extrusion (contained)	Displaced portion of NP material with a narrower base than its displaced portion extends beyond AF, but remains covered by AF or PLL	Symptomatic cases could benefit from endoscopic decompression, as persistent bulging often leads to poor response to conservative treatment; however, if nerve compression is minimal, observation may still be appropriate.
Extrusion (uncontained)	Displaced portion of NP material with a narrower base than its displaced portion extends beyond AF, but remains covered by AF or PLL	Exposure causes higher inflammatory response, causing radiculopathy; usually responsive to surgical intervention.
Sequestration	Free NP fragment displaced from extrusion site and completely separated from disc, might migrate cranially/caudally	High surgical indication due to fragment migrations and acute symptoms; localization is important in surgical planning; spontaneous resorption typically occurs within 6–12 weeks. Follow-up Magnetic Resonance Imaging (MRI) may show resolution, but residual pain may persist due to inflammatory response to NP exposure.

**Table 2:** Summary of LDH types based on anatomical location.

Anatomical Location	Preferred Endoscopic Approach	Surgical Insights & Notes
Central	Interlaminar [22]; UBE-LD [23]	Especially effective at L4–L5 and L5–S1, due to wider interlaminar window; Allows preservation of posterior elements, such as facet joints and lamina [22]; UBE provides bigger working space and has bilateral decompression capabilities [23]. The same applies to IELD, but in experienced hands.
Paracentral	Interlaminar [24]; Transforaminal [25,26,27]; UBE-LD [23,28]	Interlaminar approach is often preferred at L5–S1 due to high iliac crest [21]; The traversing nerve root is usually compressed, so precise decompression is critical to avoid residual symptoms; Annular modulation or foraminoplasty is rarely required; UBE is effective for paracentral herniations, including at L5–S1, where narrow foraminal access may limit transforaminal approaches [23,28].
Foraminal	Transforaminal with foraminoplasty [29]; Modified Transforaminal [30]; Paraspinal or contralateral UBE-LD [28]	The transforaminal inside-out technique allows early intradiscal access, outside-in technique is more effective for severe foraminal stenosis and allows for more extensive bone resection [29]; foraminoplasty allows for direct visualization of the entire neuroforamen, including the “hidden zone of Macnab” [29]; extraforaminal approach used when pathology lies lateral to facet joint [30].
Extraforaminal (Far-Lateral)	Modified Transforaminal [30,31]	Allows preservation of posterior elements, such as facet joints and lamina; Technically demanding due to limited working space and proximity to dorsal root ganglion [30].
Cranially Migrated	Interlaminar [32]; Transforaminal [33]; UBE-LD [28]	Performing a foraminotomy is usually indicated in transforaminal approach [30]; calcified disc could convert to bone resection or open surgery; UBE accesses cranially migrated herniations not reachable with TELD [28].
Caudally Migrated	Interlaminar [32,38]; Transforaminal [34,35,36,37]; UBE-LD [28]	Performing a foraminotomy is usually indicated in transforaminal approach; calcified disc could convert to bone resection or open surgery [28].

**Table 3:** Comparison of the three full-endoscopic discectomy techniques. Including TELD, IELD, and UBE/UBE-LD.

Approach	Technique Types	First Surgery	Geographic Trends
Transforaminal Endoscopic Lumbar Discectomy (TELD)	Inside-out & Outside-in [69]	Concept by Kambin (1973) [70]; first surgery by Hijikata (1975) [71]	Not specific to one approach, bibliometric analyses of FELD research show that China, South Korea, and the U.S. dominate the field, with over 80% of published studies [76].
Interlaminar Endoscopic Lumbar Discectomy (IELD)	Single Technique with minor variations: Direct-Posterior, Translaminar & Axillary/Shoulder [56,57]	First described as open interlaminar discectomy, adapted to full-endoscopic by Ruetten et al. (2006) [72,73]	Not specific to one approach, bibliometric analyses of FELD research show that China, South Korea, and the U.S. dominate the field, with over 80% of published studies [76].
Unilateral Biportal Endoscopic Lumbar Discectomy (UBE-LD)	Interlaminar, Contralateral & Paraspinal [61]	Concept by De Antoni et al. (1996) [77]; modern biportal technique first defined by Choi et al. & Eum et al. (2016) [74,75]	Primarily developed and studied in South Korea (82.4% of publications); top 10 most-cited UBE articles are all from South Korea [78].

## Data Availability

Not applicable.
